# Synchronised nesting aggregations are associated with enhanced capacity for extended embryonic arrest in olive ridley sea turtles

**DOI:** 10.1038/s41598-019-46162-3

**Published:** 2019-07-05

**Authors:** Sean A. Williamson, Roger G. Evans, Nathan J. Robinson, Richard D. Reina

**Affiliations:** 1 School of Biological Sciences, Monash University, Clayton Victoria, Australia; 2 Cardiovascular Disease Program, Biomedicine Discovery Institute and Department of Physiology, Monash University, Clayton Victoria, Australia; 3 The Leatherback Trust, Goldring-Gund Marine Biology Station, Playa Grande, Costa Rica; 4 Department of Biology, Indiana-Purdue University, Fort Wayne, Indiana USA; 5 Present Address: Cape Eleuthera Institute, Cape Eleuthera Island School, Eleuthera, The, Bahamas

**Keywords:** Evolutionary developmental biology, Animal physiology, Ecophysiology, Embryology

## Abstract

Sea turtle species in the genus *Lepidochelys* exhibit an unusual behavioural polymorphism, nesting in both aggregations and solitarily. Aggregated nesting events, termed ‘arribadas’, involve hundreds of thousands of females congregating at a single nesting beach over a few days to oviposit their eggs. Aggregate and solitary nesting behaviours are associated with distinct inter-nesting intervals, three and four weeks for non-arribada and arribada nesters respectively. Consequently, embryos are maintained in pre-ovipositional embryonic arrest in the hypoxic oviduct for different lengths of time depending on the mother’s reproductive behaviour. However, sea turtle embryos are limited in their capacity to remain in arrest and will subsequently die if held in hypoxia too long. Here, we tested whether embryos oviposited during arribada or non-arribada nesting differ in their capacity to be maintained in pre-ovipositional arrest. Olive ridley turtle (*Lepidochelys olivacea*) eggs from eight clutches (four from each nesting tactic) were divided among seven treatments after oviposition; normoxia (control; 21% O_2_), or hypoxia (1% O_2_) for 3, 3.5, 4, 8, 15 or 30 days, before being returned to normoxia. Arribada eggs were capable of extending pre-ovipositional arrest for longer, with some eggs from the 8- and 15-day hypoxia treatment still hatching while no non-arribada eggs hatched after more than four days in hypoxia. This difference in embryonic capacity to survive extended periods of arrest may be an important mechanism facilitating arribada behaviour by allowing longer inter-nesting intervals. Our finding provides an intriguing insight into the physiological mechanisms that are integral to this unique mass-nesting behaviour.

## Introduction

The evolution of synchronised reproductive behaviour has been documented in a range of animals and plants, but aggregated reproductive synchrony is unusual amongst large vertebrates (reviewed in^1^). Two well-known exceptions are those of the *Lepidochelys* sea turtle genus, which nest in large synchronised aggregations termed ‘arribadas’. Interestingly, both species of *Lepidochelys* (olive ridley turtle *Lepidochelys olivacea* and Kemp’s ridley turtle *Lepidochelys kempii*) also display behavioural reproductive polymorphism, whereby individuals can interchangeably nest in arribadas or nest solitarily in individual nesting events as other species of sea turtle do^1–3^. Arribadas can involve hundreds of thousands of turtles nesting at a single nesting beach, usually over three to four days and nights^1,4^.

A few explanations regarding the selective advantages for arribada nesting behaviour have been proposed. Nesting in large aggregations has been suggested to improve mate-finding^1,5^ in a widespread pelagic species^6^. Synchronised reproductive behaviour also offers potential fitness benefits through higher rates of multiple mating and paternity^7,8^. Arribadas have also been suggested as a mechanism to saturate predators^9–11^ in order to increase hatchling survival rate, including during early hatchling dispersal. Potential cues that stimulate arribada aggregations include meteorological (wind and rain), lunar, social, and sensory (olfaction) cues that could trigger nesting^1^. However, the proximate and ultimate causes remain to be determined.

The behavioural nesting polymorphism of olive ridley turtles is associated with different inter-nesting intervals (the period between subsequent clutches). They generally oviposit two clutches three weeks apart when nesting solitarily, or four to five weeks apart when nesting in an arribada^3,7,9,12,13^. Follicles for the subsequent clutch are ovulated one to two days after the first nest is oviposited^12,14,15^. The ova are fertilised shortly after ovulation^16,17^ and embryos have commenced development within three days of the first nesting^18,19^. Prior to oviposition all turtle embryos arrest development at the gastrulation stage^18–20^. This pre-ovipositional embryonic arrest is maintained by hypoxia (low oxygen) in the oviducts, thereby affording the mother flexibility in her nesting date^21,22^. The arrest can be artificially extended by placing eggs in hypoxia within 12 hours after oviposition^23–25^. To meet the schedule needed for synchronised nesting, ridley females nesting in arribadas are probably keeping embryos in arrest for up to two weeks longer than those nesting solitarily. Whether this putative longer period of pre-ovipositional embryonic arrest is associated with altered egg physiology, resulting in altered capacity for extended arrest, is the subject of this investigation.

We hypothesised that eggs oviposited in arribada and solitary events differ in their ability to be maintained in embryonic arrest after oviposition. It is known that turtle eggs are temporally limited in their capacity to extend arrest^24–26^. It is possible that arribada eggs have a greater ability to maintain arrest enabling these females to nest at longer intervals. Alternatively, non-arribada eggs may be able to maintain arrest for longer under experimental conditions after they have been laid because they may have spent less time in pre-ovipositional arrest in the oviduct. Here, we assessed the capacity of arribada and non-arribada eggs to be maintained in pre-ovipositional embryonic arrest, by incubating eggs in hypoxia for varying lengths of time after oviposition.

## Materials and Methods

### Regulatory approval

Experiments were performed in accordance with the relevant guidelines and regulations. Monash University’s School of Biological Sciences Animal Ethics Committee (Approval BSCI/2015/10) approved all experimental procedures. Field research was carried out under a scientific permit issued by the Costa Rican Ministerio Del Ambiente y Energía (MINAE), Sistema Nacional de Áreas de Conservación, Área de Conservación Tempisque (Resolución No ACT-OR-DR-085-15).

### Egg collection

Egg clutches were collected from eight different nesting females at Playa Ostional in the Refugio de Vida Silvestre Ostional, Costa Rica. Between 9:09 and 9:36 pm on the 26th of October 2015, four clutches of eggs (*n* = 96 to 104 eggs per clutch, 399 eggs total) were collected from nesting females that were not nesting during an arribada. The closest arribadas to this date (26th of October 2015) were between the 6th and 11th of October 2015 and the 5th and 7th of November 2015. A further four clutches of eggs (*n* = 57 to 95 eggs per clutch, 308 eggs total) were collected between 4:03 and 4:14 pm on the 7th of November 2015 from nesting females during an arribada. All clutches were collected by placing a plastic bag into the nesting cavity to catch the eggs as they were laid. The maximum time between oviposition of the first and last egg of each clutch was 18 mins. Once the last egg was oviposited the egg bag was removed from the nest and quickly (<5 mins) carried a short distance (<1 km) to the MINAE station at Ostional. All eggs were individually numbered with soft pencil and allocated to one of seven treatments. Placement of eggs into their respective treatments was complete within 40 to 100 mins after oviposition. Once all eggs were in their treatments they were driven for 2.5 h to the headquarters of Parque Nacional Marino Las Baulas for incubation.

### Experimental design

Each clutch of eggs was evenly divided between seven treatments (Table 1). The first was a normoxic control, in which eggs were randomly assigned one of three incubators (described below), within which they were placed directly into sand and kept in normoxia (~21% O_2_) until hatching. The other six treatments involved placing eggs into resealable bags (Ziploc, United States) as described previously^27^. Within each bag the eggs rested on a wire mesh above 10 mL of distilled water in a plastic container with no lid. There were between three and four separate resealable bags and containers used per treatment. Nitrogen gas (100% industrial grade; INFRA G.I., San Jose, Costa Rica) was then passed through the bag at eight L.min^−1^ for three mins through in-flow and out-flow valves, that had been inserted at each end of the resealable bag, before the valves were closed. Each bag was re-gassed as described above three times per day, for the duration of the experimental treatments. The six hypoxic treatments lasted either 3, 3.5, 4, 8, 15, or 30 d. Following the completion of each treatment period eggs were removed from their bag and placed into sand in incubators (described below). When the eggs were removed from hypoxia a subsample of eggs (*n* = 2 to 4 per treatment, 18 eggs total) were opened and staged according to Miller’s^18^ 31-stage development chronology. A subsample of the control treatment eggs (*n* = 3) were also opened and staged within two hours of oviposition.

**Table 1:** Comparison of embryonic development between treatments.

	Nesting tactic	Control	3-day	3.5-day	4-day	8-day	15-day	30-day
No. eggs	Non-arribada	71	53	55	54	56	54	56
	Arribada	48	44	44	43	44	43	42
No. eggs opened after treatment	Non-arribada	2	2	1	1	2	2	2
	Arribada	1	0	2	2	2	2	0
Embryonic stage of opened eggs*	Non-arribada	6 & 6	6 & 8	9	8	6 & 8	8 & 8	6 & 10
	Arribada	6	—	10 & 11	8 & 12	6 & 11	8 & 14	—

Olive ridley eggs were collected from non-arribada and arribada nesting females and incubated following different durations of post-oviposition hypoxia.

*Staged according to Miller’s^18^ 31-stage developmental chronology.

### Incubation

Other than when eggs were kept in hypoxia they were incubated in normoxia in beach sand (7% moisture content by mass) within incubators (GQF HovaBator model 1632; Grandview Management, Baldivis, Australia) set to 28 °C. These eggs were monitored three times per day for formation of the opaque white spot on the surface of the egg. An egg was considered to have formed a white spot once there was an opaque white circle on the surface of the egg that was obvious to the naked eye (>2 mm). Due to condensation whilst eggs were in the resealable bags it was not possible to accurately monitor the timing of formation of the white spots on eggs incubated in hypoxia. Presence or absence of a white spot was recorded when eggs were removed from hypoxia. Twenty days after eggs were removed from their respective treatments they were transported a short distance (<200 m) to a beach hatchery and buried in nest cavities that had been dug by hand to mimic the natural shape of an olive ridley turtle nest.

### Hatching and embryonic death

The first 20 hatchlings to emerge from each nest had their mass (±0.1 g) recorded using a scale and their head width, carapace length and width (all ±0.1 mm) measured using a digital calliper. Two days after first emergence of hatchlings each nest was excavated. Unhatched eggs were counted to determine the hatching success (proportion of eggs to hatch) for each treatment. The unhatched eggs were then opened and the stage of embryonic development was determined according to Leslie *et al*.’s^28^ field embryo-staging method. The method classifies Miller’s^18^ 31-stage developmental chronology into four broader stages as summarised by Rafferty *et al*.^29^.

### Statistical analysis

Cochran-Mantel-Haenszel (CMH) tests were used to assess between-treatment variation in the proportion of eggs to form white spots, form white spots in hypoxia, hatch, and die at Leslie *et al*.’s^28^ four developmental stages, whilst adjusting for nesting strategy of the mother (arribada vs non-arribada). Woolf’s test was used to check for any two-way interactions between treatment and nesting strategy for the proportions described above. Post-hoc analysis of the CMH tests was conducted by assessing between-treatment variation using pair-wise Bonferroni corrected chi-squared tests. Fisher’s exact tests were used to assess within-treatment differences in hatching success between arribada and non-arribada eggs. Two-way analysis of variance (ANOVA) was used to assess between-treatment and between-nesting tactic variation in latency (time) till white spot, aerobic latency (total time excluding time spent in hypoxia), and hatchling morphometric traits (mass, head width, carapace length and width). Post-hoc comparisons were made using Tukey’s Honest Significant Difference (HSD) test. The assumptions of homoscedasticity and normality for ANOVA models were assessed using quantile plots in the Plot package in R studio^30^. All analyses were conducted using R software^30^. Values presented are mean ± standard error unless otherwise stated. Two-tailed *p* ≤ 0.05 was considered statistically significant.

## Results

### White spot formation

There was significant between-treatment variation in the proportion of eggs that formed white spots (*X*^2^_CMH_ = 61.46, d.f. = 6, *p* < 0.0001), with no significant interaction with nesting strategy (Woolf test *X*^2^ = 0.003, d.f. = 1, *p* = 0.96). Fewer eggs in the 15-day hypoxia treatment formed white spots when compared to all other treatments, except the 30-day hypoxia treatment (Table 2). A large proportion of eggs formed white spots whilst they were still in hypoxia (Table 2), and in one case a white spot formed on the underside of an egg. For eggs that formed white spots after removal from hypoxia, there was a significant interaction between treatment and nesting tactic in the latency (time) until white spot formation (interaction term: F = 4.39, d.f. = 2, *p* = 0.01; Table 3). However, latency until white spot formation generally increased with increasing time spent in hypoxia for both nesting tactics (treatment effect: F = 3502, d.f. = 4, *p* < 0.0001; Table 3). After accounting for time spent in hypoxia, there was still a significant interaction between treatment and nesting tactic in the aerobic latency (total time excluding time in hypoxia) until white spot formation (interaction term: F = 4.39, d.f. = 2, *p* = 0.01; Table 3). Aerobic latency until white spot formation was shorter for arribada eggs in the 3-day and 4-day hypoxia treatments when compared with control (Table 3).

**Table 2:** Number of eggs in each treatment (% of total) to form white spots (WS) and number of WS (% of WS) that formed whilst in hypoxia and normoxia.

	Nesting tactic	Control	3-day	3.5-day	4-day	8-day	15-day	30-day	*P*-value*
Total WS formed	Non-arribada	69 (100%)	52 (100%)	54 (100%)	53 (100%)	53 (88.1%)	36 (69.2%)	55 (98.2%)	<0.0001
	Arribada	46 (97.9%)	43 (97.7%)	41 (97.6%)	42 (100%)	43 (97.7%)	40 (97.6%)	33 (78.6%)	<0.0001
WS formed in hypoxia	Non-arribada	N/A	45 (86.5%)	54 (100%)	34 (64.2%)	29 (54.7%)	34 (94.4%)	55 (100%)	<0.0001
	Arribada	N/A	39 (90.7%)	41 (100%)	32 (76.2%)	43 (100%)	40 (100%)	33 (100%)	<0.0001
WS formed in normoxia	Non-arribada	69 (100%)	7 (13.5%)	0 (0%)	19 (35.8%)	24 (45.3%)	2 (5.6%)	0 (0%)	<0.0001
	Arribada	46 (100%)	4 (9.3%)	0 (0%)	10 (23.8%)	0 (0%)	0 (0%)	0 (0%)	<0.0001

Olive ridley eggs were collected from non-arribada and arribada nesting females and incubated following different durations of post-oviposition hypoxia.

*Chi-squared tests were used to assess between-treatment differences for separated arribada and non-arribada data.

**Table 3:** Latency to white spot (WS) formation and first hatchling emergence between treatments.

	Nesting tactic	Control	3-day	3.5-day	4-day	8-day	15-day	30-day
Mean latency to WS (d)	Non-arribada	0.8 ± 0.0^A*^	3.5 ± 0.1^B^	—	4.8 ± 0.1^C^	8.8 ± 0.1^D^	16.0 ± 0 ^E^	—
	Arribada	1.0 ± 0.0^X*^	3.4 ± 0.2^Y^	—	4.6 ± 0.1^Z^	—	—	—
Mean aerobic latency to WS (d)	Non-arribada	0.8 ± 0.4^A*^	0.5 ± 0.1^A^	—	0.8 ± 0.1^A^	0.8 ± 0.1^A^	1.0 ± 0^A^	—
	Arribada	1.0 ± 0.0^X*^	0.4 ± 0.2^Y^	—	0.6 ± 0.1^Y^	—	—	—
Latency to first hatchling emergence (d)	Non-arribada	49	52	53	52	—	—	—
	Arribada	50	52	51	51	54	57	—

Olive ridley eggs were collected from non-arribada and arribada nesting females and incubated following different durations of post-oviposition hypoxia. The number of eggs from each treatment group that formed a white spot after incubation in normoxia are shown in Table 2. All eggs from the 3.5-day and 30-day treatment formed WSs while in hypoxia, as did the 8- and 15-day arribada eggs, so there are no data for latency to white spot. Furthermore, no 30-day eggs hatched, nor did any 8- and 15-day non-arribada eggs, so there are no data for latency to hatchling emergence. When superscript letters are the same, there was no significant between-group difference within nesting tactic according to an ANOVA and Tukey’s HSD post-hoc test. An asterisk (*) denotes a significant difference within each treatment between each nesting tactic according to an ANOVA and Tukey’s HSD post-hoc test.

### Hatching

**Figure 1:**
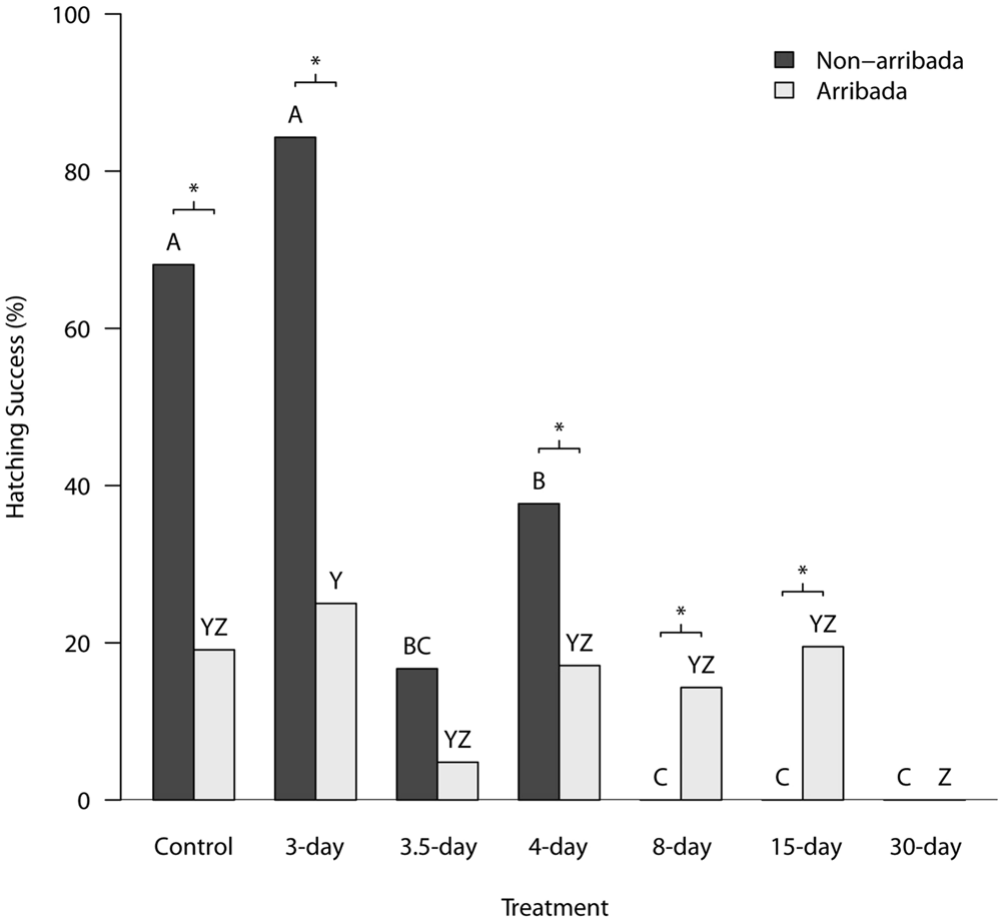
Olive ridley hatching success (%) between various treatments, partitioned according to maternal nesting tactic. Eggs (*N* = 686) were collected from either non-arribada (*N* = 387) or arribada nesting events (*N* = 299) and placed in either normoxia (21% O_2_; control), or hypoxia (1% O_2_) for 3, 3.5, 4, 8, 15, or 30 days. After their respective treatments all eggs were returned to normoxia. When letters above each bar are the same, there was no significant between-group difference for each nesting tactic in hatching success (Bonferroni corrected Chi-squared test with 21 pair-wise comparisons; p ≤ 0.05). A line and asterisk (*) above treatment group indicates significant between-nesting tactic difference in hatching success within each treatment according to a Fisher’s exact test (p ≤ 0.05).

Latency to first emergence of a hatchling from the nest varied among treatments, but the differences did not correspond directly with the duration of hypoxic incubation (Table 3). The proportion of eggs to hatch varied significantly between treatments (*X*^2^_CMH_ = 169.62, d.f. = 6, *p* < 0.0001; Fig. 1), with no interaction with nesting strategy (Woolf test *X*^2^ = 0.03, d.f. = 1, *p* = 0.86). The general trend was for a reduction in hatching success when eggs were kept in hypoxia for longer periods of time (Fig. 1). However, there were differences in hatching success between the arribada and the non-arribada eggs within some treatments (Fisher exact tests p always ≤0.05; Fig. 1). Generally, the non-arribada eggs had greater hatching success than the arribada eggs within each treatment (Fig. 1). However, after 8 or 15 days in hypoxia all non-arribada eggs failed to hatch, whereas the arribada eggs that were subjected to these two treatments had hatching success comparable to the arribada eggs from the other treatments (Fig. 1). No eggs, from either nesting strategy, hatched after 30 days in hypoxia (Fig. 1).

### Embryonic mortality

There was significant between-treatment variation in the proportion of embryos that died at each stage of development (*X*^2^_CMH_ = 143.2, d.f. = 18, *p* < 0.0001; Table 4), with no interaction with nesting strategy (Woolf test *X*^2^ = 0.01, d.f. = 1, *p* = 0.92). That is, the proportion of early stage death (Stage 0) generally increased with the duration eggs spent in hypoxia (Table 4).

**Table 4:** Comparison between treatments of the number (%) of embryos to die at each of Leslie *et al*.’s^28^ four developmental stages.

Nesting tactic	Stage	Control	3-day	3.5-day	4-day	8-day	15-day	30-day
Non-arribada	0	7 (32%)	1 (13%)	20 (44%)	10 (30%)	28 (52%)	52 (100%)	54 (100%)
	1	2 (9%)	0 (0%)	5 (11%)	2 (6%)	5 (9%)	0 (0%)	0 (0%)
	2	2 (9%)	1 (13%)	5 (11%)	5 (15%)	6 (11%)	0 (0%)	0 (0%)
	3	11 (50%)	6 (75%)	15 (33%)	16 (48%)	15 (28%)	0 (0%)	0 (0%)
Treatment comparison*		A	A	A	A	A	A	A
Total number of eggs^#^		69	51	54	53	54	52	54
Number failed to hatch (%)		22 (32%)	8 (16%)	45 (83%)	33 (62%)	54 (100%)	52 (100%)	54 (100%)
Arribada	0	12 (32%)	17 (52%)	31 (78%)	24 (71%)	24 (67%)	25 (76%)	42 (100%)
	1	8 (21%)	6 (18%)	6 (15%)	2 (6%)	3 (8%)	1 (3%)	0 (0%)
	2	7 (18%)	6 (18%)	0	8 (24%)	1 (3%)	1 (3%)	0 (0%)
	3	11 (29%)	4 (12%)	3 (8%)	0 (0%)	8 (22%)	6 (18%)	0 (0%)
Treatment comparison*		A	A	AB	AB	AB	AB	B
Total number of eggs^#^		47	44	42	41	42	41	42
Number failed to hatch (%)		38 (81%)	33 (75%)	40 (95%)	34 (83%)	36 (86%)	33 (81%)	42 (100%)

Olive ridley eggs were collected from non-arribada and arribada nesting females and incubated following different durations of post-oviposition hypoxia.

*When letters above each bar are the same, there was no significant between-group difference in hatching success (Bonferroni corrected Chi-squared test with 21 pair-wise comparisons; p ≤ 0.05).

^#^Total number of eggs per treatment excludes those deliberately opened for staging.

### Hatchling morphology

Hatchling mass (g) varied significantly among treatment groups (F = 17.24, d.f. = 5, *p* < 0.0001), and between nesting tactics (F = 132.30, d.f. = 1, *p* < 0.0001), with no significant interaction (F = 1.98, d.f. = 3, *p* = 0.12). Hatchling mass decreased with increasing time spent in hypoxia for the arribada eggs, whilst for the non-arribada eggs hatchlings were largest in the 3-day hypoxia treatment, and arribada hatchlings were usually smaller within each treatment than non-arribada hatchlings (Table 5).

**Table 5:** Mean hatchling traits for each treatment.

	Nesting tactic	Control	3-day	3.5-day	4-day	8-day	15-day	30-day
No. hatchlings measured	Non-arribada	20	20	8	20	0	0	0
	Arribada	9	11	2	6	6	8	0
Mass (g)	Non-arribada	16.7 ± 0.2^A*^	17.8 ± 0.3^B*^	16.6 ± 0.3^AB^	16.5 ± 0.2^A*^	—	—	—
	Arribada	14.3 ± 0.4^XY *^	14.8 ± 0.3^X*^	15.0 ± 1.5^XY^	12.8 ± 0.5^Y*^	13.7 ± 0.7^XY^	13.3 ± 0.3^XY^	—
Head Width (mm)	Non-arribada	14.2 ± 0.1^A^	14.4 ± 0.1^A^	14.3 ± 0.1^A^	14.3 ± 0.1^A*^	—	—	—
	Arribada	14.1 ± 0.1^XY^	14.4 ± 0.1^X^	14.3 ± 0.3^XY^	13.6 ± 0.1^Y*^	13.8 ± 0.1^XY^	13.7 ± 0.1^Y^	—
Carapace Length (mm)	Non-arribada	41.3 ± 0.3 ^A*^	41.8 ± 0.4^A*^	40.8 ± 0.4^A^	41.1 ± 0.4^A*^	—	—	—
	Arribada	39.1 ± 0.4^XY*^	40.1 ± 0.4^X*^	40.1 ± 0.1^XY^	38.1 ± 0.2^XY*^	39.1 ± 0.4^XY^	37.6 ± 0.5^Y^	—
Carapace Width (mm)	Non-arribada	34.1 ± 0.16^A^	33.9 ± 0.4^A^	33.8 ± 0.3^A^	33.9 ± 0.3^A*^	—	—	—
	Arribada	33.4 ± 0.4^XY^	34.4 ± 0.4^X^	34.8 ± 1.8^XY^	31.8 ± 0.6^Y*^	33.1 ± 0.8^XY^	31.9 ± 0.3^Y^	—

Olive ridley eggs were collected from non-arribada and arribada nesting females and incubated following different durations of post-oviposition hypoxia.

When superscript letters are the same, there was no significant between-group difference within nesting tactic according to an ANOVA and Tukey’s HSD post-hoc test. An asterisk (*) denotes a significant difference within each treatment between each nesting tactic according to an ANOVA and Tukey’s HSD post-hoc test.

Hatchling head width (mm) also varied significantly between treatment groups (F = 5.19, d.f. = 5, *p* < 0.001), and between nesting tactics (F = 6.47, d.f. = 1, *p* < 0.05), with a significant interaction (F = 2.66, d.f. = 3, *p* = 0.05). Post-hoc analysis showed that head width varied little across treatments for the non-arribada eggs (Table 5). However, it differed amongst treatments for the arribada eggs, with a trend for narrower heads with increased time spent in hypoxia (Table 5). Non-arribada hatchlings only had larger head width within the 4-day treatment (Table 5).

Hatchling carapace length (mm) also varied significantly among treatment groups (F = 9.51, d.f. = 5, *p* < 0.0001), and between nesting tactics (F = 41.72, d.f. = 1, *p* < 0.0001), with no significant interaction (F = 1.26, d.f. = 3, *p* = 0.29). According to a Tukey’s post-hoc test carapace length decreased with increasing time spent in hypoxia for the arribada eggs, whilst for the non-arribada hatchlings carapace length did not differ significantly across treatment. The arribada hatchlings were usually smaller within each treatment than non-arribada hatchlings (Table 5). Carapace width (mm) showed a similar pattern to carapace length (Table 5), with significant between-treatment variation (F = 3.79, d.f. = 5, *p* < 0.01), no significant variation between nesting tactics (F = 2.22, d.f. = 1, *p* = 0.13), and a significant interaction (F = 4.05, d.f. = 3, *p* < 0.01).

## Discussion

Olive ridley females that nested during an arribada oviposited eggs that had a lower hatching success than eggs from non-arribada nesters under control conditions. However, our findings indicate that embryonic arrest could be extended in eggs of females that nested during an arribada for longer than eggs from those that did not nest during an arribada. Some arribada eggs still successfully hatched after 15 days in hypoxia, whereas non-arribada eggs were only capable of surviving to hatch after four days in hypoxia. To our knowledge, this is the first finding of a developmental difference between the eggs of arribada and non-arribada nesting females.

Our finding that arribada eggs had a greater ability to extend arrest than solitary eggs provides support to the suggestion that females nesting solitarily or in arribadas also differ physiologically, potentially in relation to nutrition, age, and size of the turtle^3^. Possibly less mature, or older, or physiologically compromised females are unable to produce eggs capable of maintaining embryonic arrest for as long as females that are at their physical and reproductive performance peak. Nesting in an arribada requires increased oviducal egg retention because the inter-nesting period is longer^1,3,7,12^. Furthermore, it has been reported that when the inter-nesting period between arribada events is sufficiently extended, turtles that nested in the first arribada will start to change behaviours and nest solitarily before the second arribada event commences^3^. However, we did not know the inter-nesting interval of the mothers so cannot rule out the possibility that the non-arribada females may have simply been late or early for an arribada. Clearly though, the ability to maintain pre-ovipositional embryonic arrest is critical for the evolution of this fascinating strategy.

Generally, the eggs from the non-arribada nesters had greater hatching success than those of arribada nesters, although this difference was only statistically significant for the control, three- and four-day hypoxia treatments. The reduced hatching success, even in the control arribada eggs, was not due to infertility because embryos were present. But it could be due to the longer period that we assume they spent in pre-ovipositional embryonic arrest in the mother’s oviducts prior to the arribada and oviposition^12^. It is generally known that greatly extended periods of arrest reduce hatching success^24,25^. Furthermore, olive ridley nests at arribada beaches generally have much lower hatching success; <35%^31^, than at beaches with solitary nesters; >75%^13^. However, there was much greater hatching success for arribada oviposited clutches in a study where they were moved to hatchery nest sites treated to reduce fungal and bacterial abundance^32^. Perhaps, in our current experiment, the arribada eggs were less tolerant than non-arribada eggs to the transportation back to the laboratory, which would explain why even the control eggs had lower hatching success. Further investigation of the development of arribada and non-arribada eggs (from both arribada and solitary-only beaches) under ideal incubator conditions is warranted to ascertain differences in development.

The decreased hatching success of hypoxic treatments, large proportion of eggs that formed white spots whilst in hypoxia, and the formation of a white spot on the bottom of an egg, suggests that the embryos ability to maintain arrest is compromised by prolonged hypoxia, regardless of the reproductive tactic of the mother. For the eggs that were capable of maintaining arrest, white spots then formed within one day of exposure to normoxia. Our findings also provide evidence that even if a white spot forms during hypoxic incubation, indicating the breaking of arrest, it is still possible that the egg will hatch once it is returned to normoxia, at least in this species. However, we also found that the majority of eggs in the longer hypoxic treatments formed white spots even though hatching success was reduced. Whilst white spot formation usually indicates a developing embryo, our findings provide further evidence that formation of a white spot does not always indicate that development is successfully occurring. Indeed, the process may sometimes be affected by passive abiotic factors^23^.

There are four possible explanations for why so many eggs formed white spots whilst in hypoxia. Firstly, the eggs may have been exposed to normoxia for too long prior to being placed in their respective treatments. However, 40–100 minutes of exposure to normoxia prior to placement into hypoxia would be unlikely to cause pre-ovipositional arrest to break. We previously found that it takes more than 12 hours of exposure to normoxia for arrest to be broken in green sea turtles^23^. Furthermore, in a different study on olive ridley turtles we placed eggs into hypoxia after 20–50 minutes of exposure to normoxia and this did not cause pre-ovipositional embryonic arrest to be broken^27^. If the embryos in this study did indeed break from the pre-ovipositional embryonic arrest, and then re-entered embryonic arrest once placed back into hypoxia, this would be the first such finding reported.

A second explanation could be that the treatments may not have maintained sufficiently low oxygen tension over time. We are confident that the level of oxygen within each bag remained low (<1.5% O_2_) because the bags take 16–24 hours to reach (2–3% O_2_) and they were re-gassed approximately every 8 hours. A third explanation could be that eggs were able to break from arrest and recommence development even though they were maintained in hypoxia. This conclusion is supported by the observation that at least one of the embryos that were staged after incubation in hypoxia had proceeded beyond Miller’s Stage 6. Nevertheless, it is unlikely that embryos would be able to develop normally initially without sufficient oxygen availability, unless developmental rate was reduced. This would be the first such finding of embryos surviving to hatch after development in hypoxic conditions. Fourthly, as mentioned previously, white spot formation might not necessarily be indicative of normal embryonic development occurring and the process may be affected by passive abiotic factors^23^. Regardless, the fact that a large proportion of eggs formed white spots whilst in hypoxia, irrespective of nesting tactic, does not undermine the major conclusion we have made from our findings, that the ability to maintain embryonic arrest differs according to reproductive strategy.

The difference in hatchling size between treatments was unexpected. Although, the biological significance of our findings is hard to determine because there was no linear trend in hatchling size with increasing time spent in hypoxia. Potentially, extended embryonic arrest reduces the time available for development and/or reduces the capability of the embryo to assimilate egg resources. We found support for the former, because the time until first emergence of hatchlings from the nest was not delayed by a length of time equivalent to the time the eggs spent in hypoxia (i.e. embryos caught up some of the delayed development). This suggests there may be important ecological ramifications if mothers maintain eggs in arrest for longer periods through increased inter-nesting intervals. Extended embryonic arrest has been shown to impact hatchling morphology and fitness in the flatback turtle^24^. Alternatively, the difference could be due to any abiotic differences that eggs from each treatment experienced once they were relocated to their respective nests in the hatchery. Furthermore, the differences could be a result of mostly, or only, smaller eggs from smaller females surviving to hatching in the longer hypoxia treatments.

We also found a difference in hatchling size between arribada and non-arribada eggs. However, this may be an artefact of the mother’s size. It is well established that hatchling size is related to egg and female size, with larger females ovipositing larger eggs which in turn produce larger hatchlings^33–37^. Therefore, the difference we found could have been an artefact of differences between initial egg size and females. However, we were unable to ascertain maternal identity of hatchlings in the current study, so were unable to assess between-female hatchling size variation here. Furthermore, we were unable to measure initial egg mass for each egg as this would have considerably delayed placement of eggs into hypoxia. Future investigation of the impact of arribada and solitary nesting behaviours on ridley hatchling morphology and fitness is warranted^1^.

In conclusion, we found differences in egg development between females that nested in arribada and non-arribada events. Arribada-laid eggs had lower hatching success when incubated in normoxia or for short periods (≤4 days) in hypoxia, but were paradoxically capable of maintaining pre-ovipositional embryonic arrest for longer. From our data we think that pre-ovipositional arrest is an integral mechanism enabling ridley turtles to synchronise arribada nesting behaviour. Our findings provide new information on this interesting reproductive tactic and our understanding of its evolution and ecological implications. Future research is warranted to further investigate developmental processes that allow this unique reproductive tactic to occur.

## Data Availability

Data available from the Dryad Digital Repository: 10.5061/dryad.4s600ch.
